# The Roles of Communication, Collaboration, and Social Support on Dyadic Mental Health in the Context of Chronic Pain

**DOI:** 10.1093/geroni/igaa057.2233

**Published:** 2020-12-16

**Authors:** Karen Lyons

**Affiliations:** Boston College, Chestnut Hill, Massachusetts, United States

## Abstract

Drawing on the Theory of Dyadic Illness Management, the study examined the roles of communication, collaborative decision-making and social support on the mental health of 177 couples living with chronic pain. Couples ranged in age from 26-81 years of age; mean age for partner with chronic pain = 55.01 (SD=11.53) and partner without chronic pain = 57.45 (SD=12.50). Using multilevel modeling and controlling for pain severity, pain interference, time since diagnosis, age, shared activities and relationship quality, communication and collaborative decision-making played significant roles in predicting mental health of both members of the couple. Comparative dyadic analysis showed that couples with optimal dyadic mental health had significantly better communication, less concealment, greater collaboration and greater levels of support than couples with poor or incongruent dyadic mental health. Discussion will center on the roles of collaborative illness management behaviors in optimizing dyadic mental health in the context of chronic illness. Part of a symposium sponsored by Dyadic Research on Health and Illness Across the Adult Lifespan Interest Group.

